# Microwave Assisted Extraction of Raw Alginate as a Sustainable and Cost-Effective Method to Treat Beach-Accumulated *Sargassum* Algae

**DOI:** 10.3390/polym15142979

**Published:** 2023-07-08

**Authors:** Aleksandra Nesic, Maria Valeria De Bonis, Giovanni Dal Poggetto, Gianpaolo Ruocco, Gabriella Santagata

**Affiliations:** 1 Vinca Institute of Nuclear Sciences, University of Belgrade, Mike Petrovica Alasa 12-14, 11 000 Belgrade, Serbia; 2 College of Engineering, Campus Macchia Romana, University of Basilicata, 85100 Potenza, Italy; mv.debonis@gmail.com (M.V.D.B.); gp.ruocco@gmail.com (G.R.); 3 National Council of Research, Institute for Polymers, Composites and Biomaterials, Via Campi Flegrei 34, 80078 Pozzuoli, Italy; giovanni.dalpoggetto@icpb.cnr.it (G.D.P.); gabriella.santagata@ipcb.cnr.it (G.S.)

**Keywords:** alginate, microwave-assisted extraction, *Sargassum* algae

## Abstract

This paper highlights the potential of *Sargassum* algae, recovered from raw beach seaweed wastes, as a valid source of valuable sodium alginate. Alginate is a biodegradable, highly attractive polysaccharide widely used in food, pharmaceuticals, and biomedicine applications. The aim of this work is to employ a new eco-sustainable and cost-effective extractive method to obtain alginate as a raw material from pollutant organic *Sargassum* seaweeds. Algae were exposed to microwave pre-treatment under static and dynamic conditions, and three different extractive protocols were followed: (a) conventional, (b) hot water and (c) alkaline method. All samples were characterized by GPC, SEM, FTIR/ATR and TGA. It was found that alginate’s best performances were obtained by the microwave dynamic pre-treatment method followed by alkaline extractive protocol. Nevertheless, the microwave pre-treatment of algae allowed the easiest breaking of their cell walls and the following fast releasing of sodium alginate. The authors demonstrated that microwave-enhanced extraction is an effective way to obtain sodium alginate from *Sargassum*-stranded seaweed waste materials in a cost-effective and eco-sustainable approach. They also assessed their applications as mulching films for agricultural applications.

## 1. Introduction

*Sargassum* algae are brown free-floating seaweed belonging to the *Phaeophycean* family. They can be found worldwide in maritime temperate and tropical regions, providing refuge, shelter, and food for many animal species, such as sea turtles and shrimps. In recent years, their wide diffusion ashore has gone out of control, even to the point of forming dense clumps of slowly beach-fouling rotting weeds, yielding toxic solid waste alongside urban beaches. In the United States, beach fouling has become a major problem in some urban areas, particularly along the Gulf Coast. Recently, the territory of the Virgin Islands declared a state of emergency due to an unprecedented bloom of seaweed along its coast, which caused widespread beach-fouling and posed a serious risk to public health [1]. Indeed, when developing as harmful algae blooms, this phenomenon can cause serious problems to coastal traffic of boats and swimmers up to fish survival, since they strongly deplete water oxygen, light and space on the sea floor [2,3]. Nevertheless, these harmful brown seaweeds represent a valid source of alginate, a well-known and exploited biopolymer, which plays a key role as a structural component of the plant seaweeds. Actually, alginates are designed to be the seaweed’s main skeletal compound since their hydrogels, located in the intercellular walls of the algae, are responsible for the mechanical strength and flexibility required to withstand the force of the water in which the seaweeds grow. In addition, they play a healthy role in protecting seaweeds against environmental stressors and sea pathogens [4,5].

**Figure 1:**
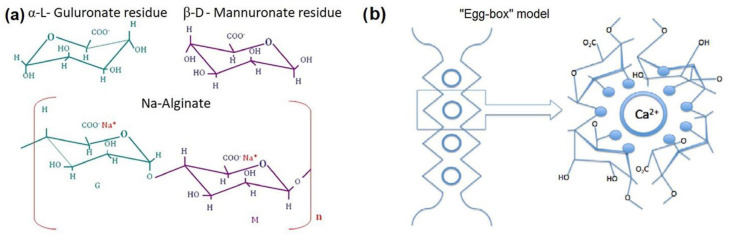
(**a**) Alginate chemical structure and (**b**) “Egg-box” model.

In the cell tissue, alginates are mainly found as insoluble gels of mixed calcium, magnesium, sodium and potassium salts; they are extracted from the grounded thallium upon the collapse and subsequent transformation of their tissue into a brown mass [6]. Actually, alginic acid is a complex mixture of oligo-polymers, derived from the polymerization of two types of monomers, β-D-mannuronic acid (M) and α-L-guluronic acid (G), linked through glycosidic junction points between the C1 and C4 positions of adjacent monomers. Thus, a linear chain of repeating units (polymannuronic acid (MM), polyguluronic acid (GG) and a mixed polymer (MG) including also sequences like GGM and MMG are formed. In particular, the mannuronic acid forms linear and flexible conformation due to the β (1–4) linkages; whereas the guluronic acid gives rise to α (1–4) linkage, introducing in this way a buckled and folded structure able to develop steric hindrance around the carboxyl groups. For this reason, the G-block segments provide rigid conformations responsible for a pronounced stiffness of the macromolecular chains. The ratios of the three types of blocks, MM, GG and MG, determine the physical properties of alginates since alginates with high M content evidence higher viscosity [3,4], whereas alginates with high G content show higher gelling properties in the presence of divalent cations, developing the form of well known “egg-box” structure (see Figure 1). The earlier statements reflect the enormous applicative versatility of the polymer, strictly dependent on the guluronate residues content and on the average number of consecutive guluronate moieties in G-blocks structures and the molecular mass weight distribution. The resulting polysaccharide, alginate, is biodegradable and biocompatible and can be widely used in food, pharmaceutical and biomedical applications, due to their emulsifying, stabilizing, and gelling properties [7].

The purpose of this work is to employ new eco-sustainable and cost-effective extractive methods to obtain alginate from *Sargassum* raw seaweed waste and to use it as a biopolymer matrix for agricultural mulching films. Finalized to this specific aim, sodium alginate extraction and purification do not need to be pushed up to the extremely high level of purity required by pharmaceutical standards. Indeed, for agricultural applications, the retaining of protein, cellulose fractions, fibers and colored pigments, represents an added value in terms of agronomic and mechanical performances of the mulching films on the soil [8].

When dealing with sodium alginate extractive methods, conventional protocols involve the use of aggressive organic chemicals for the removal of polyphenols and bleaching, large volumes of hot water, dilute acid, dilute alkali and long extraction times. As a consequence, final alginate properties are strongly influenced by the use of aggressive extractive methods, sometimes leading to polymer chemical degradation [9,10]. Therefore, conventional methods need to be suitably substituted or synergized with sustainable extractive methodologies, allowing for the optimal conditions to obtain high extraction yields and high-performing output. In the last decade, mechanical and physical techniques, such as microwave-assisted extraction (MAE), have been successfully applied to extract several biologically active compounds from a wide variety of natural resources [11,12]. Generally, the compounds obtained through MAE enjoy similar or better yields in comparison with conventional extraction processes, using less energy, time and solvent volume, thus resulting in a more eco-sustainable process. Up to date, there are only a few papers reported in the literature related to the use of the MAE approach to extract alginate from different algal species (*Ascophyllum nodosum* [13,14], *Nizimuddinia zanardini* [15] and *Saccharina latissima* [16]) and isolation of macromolecular fractions from targeted *Sargassum* algae. In fact, MAE has been investigated for the isolation of polyphenols [17,18] and fucoidan polysaccharide [19,20] from *Sargassum* algae, but to the best of our knowledge there is only one report of MAE application to extract alginate from this algae family. In this particular paper, the power level (70; 80; 90 and 100%) and microwave exposure time (between 15 and 18 min) were optimized for the extraction of alginate [21]. Framed in this context, in the present paper, recovered *Sargassum* algae wastes, have been used in order to extract sodium alginate by both the conventional protocol and a mild MAE method, in which power density and duration were optimized. In order to enhance the exposure to microwave radiation and minimize the exposure time, a specially designed laboratory-scale fluidized bed was used in the microwave cavity. Fluidized bed technology is long known for its favorable features leading to proper uniformity treatment [22,23]. In this work, the microwave extractive approach combined with a fluidized bed for the isolation of polysaccharides from the biomass is reported. Two different regimes were tested on the quality of extracted alginate: static conditions where dry algae were exposed to the specific power and microwave radiation, and dynamic conditions where algae hydrated in water were introduced to a fluidized bed in a microwave cavity at the same operating condition as static ones (the same power and exposure time). The use of the new MAE extractive method could provide an eco-sustainable and cost-effective approach for the one-pot extraction of alginate including proteins, minerals and colored pigments. The whole components were used to prepare alginate-based films for agricultural practices such as mulching. Spectroscopic (FTIR-ATR), morphological (SEM), thermal (TGA) and structural (GPC) analyses were performed in order to validate the efficiency of the MAE method and the potential usage of extracted alginate films in agriculture applications.

## 2. Materials and Methods

### 2.1. Chemicals

*Sargassum* algae were kindly supplied from Lianyungang Zhongda Seaweed Industrial Co., LTD (Lianyungang, China). The seaweed was rinsed thoroughly in fresh water to remove sediments and surface contamination. The seaweed was dried in an oven under vacuum at 40 °C for 24 h and stored in a refrigerator at 4 °C until further use. The chemicals used in this study are hydrochloric acid, sodium carbonate, absolute ethanol, acetone (purchased from Sigma Aldrich, Italy) and commercial alginate (purchased from Lianyungang Zhongda Seaweed Industrial Co. Ltd., China).

### 2.2. Pre-Processing by Microwave Exposure

**Figure 2:**
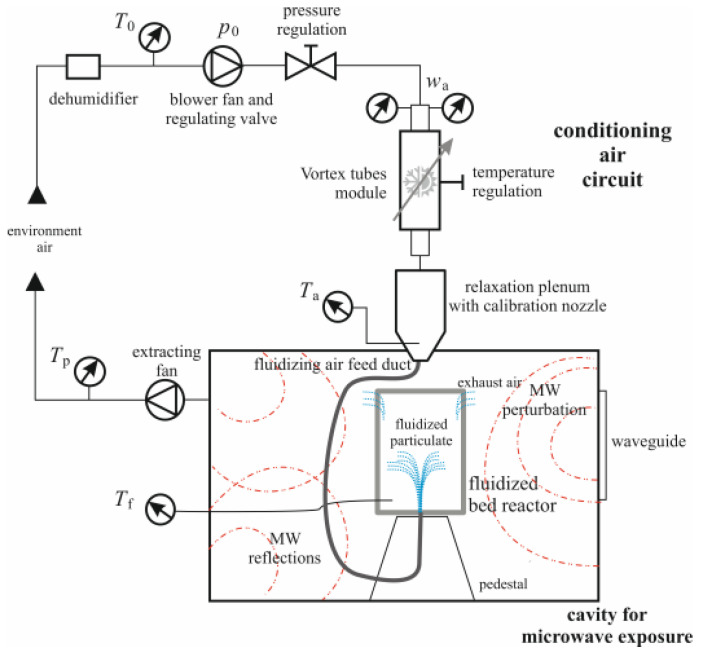
A scheme of the rig for the microwave bed.

The microwave exposure experiments were conducted in triplicates in a laboratory prototype, allowing for the enforcement of optimal power density, duration, thermal regime of working air and exposure uniformity [24,25]. To optimize the exposure to microwave radiation, a laboratory-scale fluidized bed was exploited by inserting it in the microwave cavity. The rig consisted of a process cavity equipped with a system of psychrometric air control (Figure 2). The process air was dehumidified and pressurized (Ceccato, mod. CDX9 and S4 Fonolife, Brendola, Italy) to be injected at the bottom of a fluidized bed reactor, and placed in the cavity. Air was pressure-regulated manually, to come up with the desired flow rate at the nozzle outlet way, and read by two flow meters (CS Instruments, mod. VA500, Harrislee, Germany) on two parallel branches. The working air stream was sent to the two-flow branch through a battery of three Ranque-Hilsch vortex tubes (AiRTX, Cincinnati, OH, USA), each one related to a different nominal (optimal) flow rate. By means of related valves, the air was streamed through the most appropriate tube of the battery to approximately minimize the entropy production inherent to the irreversible thermal conditioning in the vortex tube, which emerged as a spurious tube heating after a several runs. At the battery exit, therefore, either flow branch (carrying a hot or cold air stream) could be operated by automated valves whose operation allows to mix the air up to the desired thermal regime Ta (monitored by a dedicated resistance thermometer (Elsi, Lainate, Italy). Before reaching the process cavity, the air was ducted to an insulated relaxation plenum attached to a nozzle and to the distribution plate at the bottom of a glass fluidized bed reactor, by means of a silicon tube. The nozzle was flush mounted through the cavity ceiling by means of a proper electromagnetic gasket.

The distributor plate at the bottom of the reactor, and the reactor’s top lid (which included proper apertures for exhaust air) were held in place by Teflon flanges. The reactor was held in place by a rigid wired pedestal, and an optical fiber thermometer fed by a signal conditioner (Ipitek, mod. LT-X5 and LT-X5O, Carlsbad, CA, USA) was inserted at the reactor bottom, to read the fluidization temperature *T*_f_. The data were acquired by a service computer. The process cavity had a net capacity of 0.022 m^3^ and was provided by a MW waveguide and magnetron group (Samsung Electronics Italia, mod. CM1039, Cernusco s.N., Italy) producing a fixed MW power level of 991 W, whose measurement was ensured by using a power clamp meter (Fluke, mod. 345, Everett, WA, USA). To avoid air and vapor pressure build-up in the cavity proper, an exhaust balancing fan was provided to the lateral grille, normally intended to vapor discharge, while the grille on the magnetron side was sealed up. The exhaust fan also provided the air flow seeping through the interspace between the external side of the cavity wall and the cavity’s external case, in order to avoid overheating of the magnetron group. Different MW power levels were provided by insertion of carefully weighted buffer water quantities, placed in the cavity. Different working times were measured by means of a service stopwatch that drove the ON/OFF switch of the magnetron power supply. The effective MW exposure time is set by imposing a magnetron working duration Δ*t*_ON_, followed by a resting or relaxation duration Δ*t*_OFF_. 109 Several “cold” tests (i.e., with no exposure to microwaves of the fluidized bed) were conducted to ascertain the optimal fluid regime for proper fluidization, hence, to offer a uniform exposure to microwaves.

### 2.3. Alginate Extraction

Dried chopped *Sargassum* particles (particle size between 50–80 mm) underwent three different polysaccharide extraction methods. The first one was performed following the traditional protocol, involving a demineralization process in dilute acid solutions (0.1 M HCl) for 24 h at room temperature and speed of 200 rpm, and following polymer solubilization in alkaline medium (3 wt% Na_2_CO_3_) for 3 h at 90 °C and speed of 200 rpm. The second and third ones consisted of a previous exposure of dried chopped substrate to controlled microwave energy in a dedicated prototype, allowing to enforce optimal power density and duration under static and dynamic conditions [25]. Static conditions considered the use of dry algae in a microwave exposed to the energy of 3 W/g for 30 min, with Δ*t*_ON_/Δ*t*_OFF_ set up 10/50 s. The dynamic conditions correlated to the microwave procedure under the same operative conditions, but this time algae in a wet state, i.e., algae immersed in water, were exposed to the microwave chamber. The mass flow rate of biomass ranged between 0.6 and 0.8 g/min. The pre-treated raw algae particles were then subjected to alginate extraction by using three different routes: (a) hot water treatment for 3 h at 90 °C and 200 rpm; (b) conventional method as described above and (c) alkaline hot treatment (3 wt% Na_2_CO_3_) for 3 h at 90 °C and 200 rpm. Following extraction methodologies, the solutions were precipitated in absolute ethanol, washed once in acetone for 30 min and dried. All extracts were further dissolved in water at a constant concentration (1 wt%), poured on Petri dishes, and allowed to dry, in order to obtain alginate-based films.

### 2.4. Yield of Extracted Alginate

The sodium alginate yield (%) obtained in this research plan is calculated using the following equation:

$$Yield\;\left(\%\right)=\frac{mass\;of\;dry\;alginate}{mass\;of\;dry\;algae}\times 100$$

### 2.5. Morphological Analysis of Samples

Morphological analysis of raw and treated substrate samples was performed by means of an FEI Quanta 200 FEG Scanning Electron Microscope (SEM) (Hillsboro, Oregon, USA). SEM analyses were performed at room temperature, in high vacuum mode, using a large field detector (LFD) and an acceleration voltage of 20 kV. Prior to the observation, the sample surfaces were coated with a homogeneous layer (18 ± 0.2 nm) of Au-Pd alloy by means of a sputtering device (MED 020, Bal-Tec AG, Maschinenbau, Switzerland).

### 2.6. GPC

Gel permeation chromatography was performed using a GPC Max Viscotek equipped with a TDA 305 composed of refractive index (RI), low angle light scattering (LALS), right angle laser light scattering (RALS), and viscometer (IV) detectors. The column set consisted of a pre-column TSK PWXL and TSK Gel GMPWXL. All the samples were dissolved up to a concentration of ≅0.5–0.7 mg/mL and eluted in MilliQ water containing 0.2% NaN_3_ and 0.1 M NaNO_3_ to avoid any polymer agglomeration phenomena. After complete dissolution, samples were filtered through a 0.22 μm CA filter. The injection volume was 100 μL and the flow rate was 0.5 mL/min. The measurements were performed in duplicate at 40 °C, according to the temperatures of the columns and detectors.

### 2.7. FTIR/ATR

Attenuated Total Reflection Fourier Transform Infrared (FTIR-ATR) spectroscopy was carried out on dried films. The spectra were collected on a Perkin–Elmer Spectrum 100 spectrometer (Waltham, MA, USA), equipped with a Universal ATR diamond optical crystal and ZnSe focusing elements sampling accessory. All the samples were analyzed at room temperature in the range of 4000–650 cm^−1^, recorded as an average of 16 scans with a resolution of 4 cm^−1^. Before testing, all samples were dried in an oven at 60 °C for 24 h.

### 2.8. Thermal Analysis

Thermogravimetric analyses (TG) were performed by using a thermogravimetric analyzer Mettler-Toledo TG-SDTA 851 thermobalance, equipped with a differential thermal analyzer instrument. About 5 mg of samples were placed in an open ceramic crucible and heated from 25 °C up to 600 °C at a speed rate of 10 °C/min, under a nitrogen flow of 30 mL/min. Before the tests, a blank curve was measured and subtracted from the single thermograms, to correct instrumental drift [26].

## 3. Results and Discussions

### 3.1. Extraction Yield

The different extraction and pre-treatment methods of alginates with sample codes and obtained alginate yields are presented in Table 1. The yield of untreated alginate obtained by using conventional methods (demineralization and alkaline treatment) is 32%. This result is expected, since it is known that a high yield of alginates can be obtained by iterating demineralization and alkalinization processes to push the extraction up to high yields, depending on extraction time and temperature [27,28,29,30]. However, this method requires a large amount of solvents, high energy consumption and very long extraction times. Actually, this procedure leads to a very neat polymer necessary due to the expected final applications in food, pharmaceuticals, and biomedical sectors. As previously mentioned, with regard to applications in agriculture, alternative, eco-cost-effective extraction routes can be carried out. Preliminary results showed that alginate could not be extracted in water from untreated algae. On the other side, microwave pre-treatment allowed the breakup of algae cell walls and the easier release of alginate in water (samples A2 and A5). However, the extraction yield is below 5%. Rostami et al. also obtained the lowest yield of alginate for the water-treatment (3.8%) [31]. The amount of extracted alginate from pre-treated algae increased up to 20–24% when the alkaline extraction method was used, and to 32–36% when the acid+alkaline method was used. This result was in some way expected since it is well known that acid treatment represents an essential step in the extraction of alginate, because it converts alginate-magnesium or calcium salts into alginic acid, thus allowing easier solubility and conversion into water-soluble sodium-alginate during the following alkaline extraction process [10]. On the other side, when only alkaline treatment is used, the extraction of alginate occurs through an ion-exchange reaction, with reduced solubility of alginate in extractive solution. Regarding microwave pre-treatments, the dynamic condition provides a slightly higher release of alginate from the cell walls than static conditions. The obtained yield values are similar to those reported in the literature for alginate MAE from *Ascophyllum nodosum* [13] and for conventional extraction method from Madagascan brown algae [32], but higher from ultrasound-assisted alginate extraction and alginate alkaline extraction from *Sargassum* algae (13–15%) [33,34].

### 3.2. SEM

**Figure 3:**
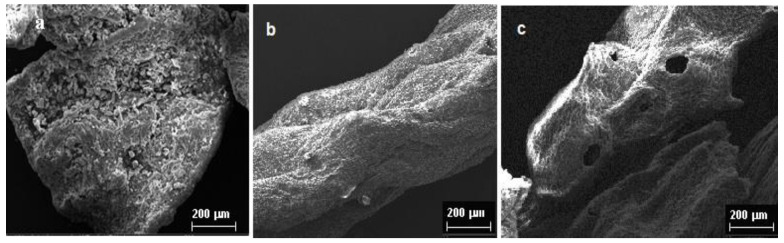
SEM micrographs of: (**a**) untreated algae, (**b**) microwave pre-treated algae under static conditions and (**c**) microwave pre-treated algae under dynamic conditions.

The micrographs of raw (a) and microwave-treated algae surfaces under static (b) and dynamic (c) conditions are shown in Figure 3. SEM micrograph of raw algae (a) reveals a rough and uneven surface, with the presence of large and irregularly spread domains. Deep morphological changes could be detected in seaweed surfaces after controlled microwave treatments (Figure 3b,c). Namely, the surface became more smoothed, homogeneous and characterized by the presence of interwoven, jagged fibrous domains. This morphological structure was preserved also on the surface of algae pre-treated under dynamic conditions. Anyway, in this case, some regular-shaped holes, probably due to water evolution previously entrapped in the cell walls matrix, are observed. Actually, it is purported here that microwave energy, penetrating into the material structure, produced an intense volumetric heat source, due to molecular friction of both polar solvents and conductive migration of dissolved ions. As a consequence, the water molecules evaporate easily, breaking the external vegetable tissues and probably inducing a sort of ion exchange, responsible for subsequent sodium alginate extraction by means of the sole mild extraction method (hot water) [35].

**Figure 4:**
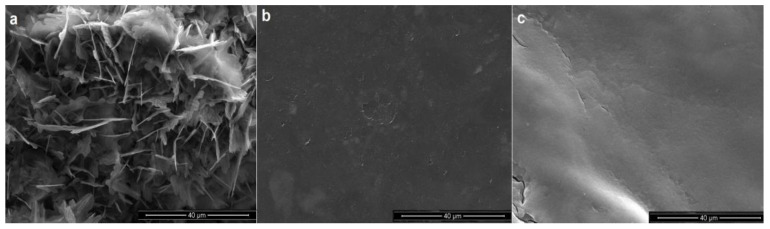
Surface morphology of (**a**) A6, (**b**) A5 and (**c**) A7 samples.

In Figure 4, the SEM morphology of alginate surface films is reported. In order to not overburden the paper, only one micrograph has been reported and discussed for each kind of extraction procedure. Specifically, A6, A5 and A7 film surfaces of alginates extracted after dynamic microwave exposure pre-treatments have been discussed. From the analysis of the A6 sample (Figure 4a), it is worth highlighting a very wrinkled and rough surface with the presence of thin and high polymer edges and holes likely associated with the presence of some fibers physically engaged to the polymer matrix and to polymer-salts complexes formed during the solvent casting of the polymer. Actually, it is very likely that some raw and native components, like cellulose fibers and minerals strictly linked to brown seaweeds, could remain unaffected in the conventional method extracted polymer since the occurring extraction procedure is intentionally not driven up to extreme purity and quality levels [36]. Regarding A5 and A7 sample micrographs related to alginates extracted in hot water (Figure 4b) and alkaline solutions (Figure 4c), it is possible to highlight substantially different surfaces, more regular, smoother and homogeneous, likely due to the peculiar extraction procedures able to provide a tight structural packing organization with a good macromolecular interconnection.

### 3.3. GPC

**Figure 5:**
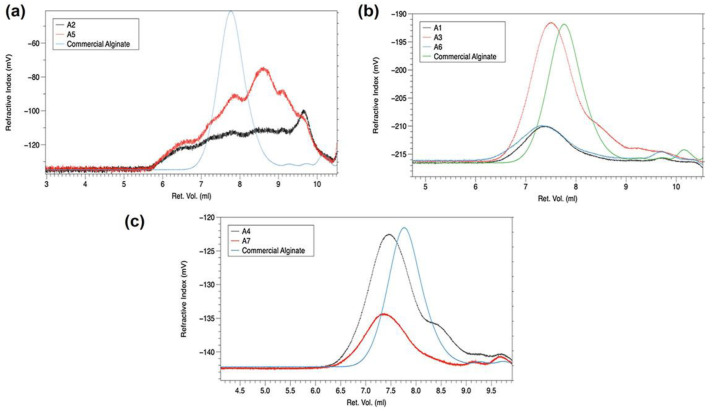
GPC chromatograms at Refractive Index detector of alginates obtained by: (**a**) hot water treatment, (**b**) conventional method, and (**c**) alkaline method.

The molecular weight distributions of all extracted alginates are presented in Table 2, and the chromatograms have been reported in Figure 5. GPC analysis was also performed on commercial alginate, used as a reference. The first attempt was to investigate the extraction of alginate from untreated and microwave-pre-treated algae in hot water. Alginates extracted in hot water (samples A2 and A5) elute as multimodal distribution with very broad polydispersity, indicating an extraction of different polymer fractions (see Figure 5a). GPC chromatograms of all alginates extracted by conventional and alkaline method displays one main broad peak and in A3 and A4 with one small shoulder (see Figure 5b,c). Microwave pre-treatment under static conditions appears to negatively influence the quality of obtained alginate, which is evidenced by lower values of Mw and η, in comparison to the untreated algae. On the other side, when microwave dynamic conditions are applied to algae, the alginates extracted by the conventional method are with higher viscosity (6.94 dL/g) and molecular weight (458,570 Da) than those obtained from untreated algae and microwave-pre-treated algae under static conditions. It appears that microwave treatment under static conditions (dry algae used) is a more aggressive approach, causing not only the change in morphology but also inducing the start of degradation of polymer units. There are some reports in the literature, that at certain conditions (time, power, etc.), microwave irradiation can induce the depolymerization of alginate, but also of other polysaccharides [37,38,39]. Apparently, the presence of water and algae in a microwave chamber (dynamic conditions) promotes an easier release of polymer fractions from cell walls but minimizes the side effects of the microwave regime.

The data in the literature show a broad spectrum of Mw and η values for extracted alginate, which is probably the consequence of different algae species, different extraction methods and extraction parameters. The values of Mw obtained in this work are higher than for alginate produced from *Sargassum* algae by ultrasound-assisted extraction followed by a demineralization process in lemon juice and an alkaline process in Na_2_CO_3_ (80–112 kDa) [40]. On the other side, Santagata et al. obtained alginates of a similar Mw range (262–438 kDa) as in this work, by varying the extraction time and acid/alkaline conditions during ultrasound-assisted extraction of alginate from *Sargassum* algae [41]. The viscosity values obtained in this work are in the range between 1.98 and 6.9 dL/g, which are similar to those reported for alginates extraction from *Sargassum* algae by alkaline method varying operative parameters (range between 1 and 6 dL/g) [42,43]. On the other side, the conventional extraction method provided alginates with viscosity values in the range between 4 and 15 dL/g [44,45].

Nevertheless, all extracted alginates in this work have higher Mw than commercial alginate, whereas all samples except hot water treated samples and A3 sample have higher viscosity, suggesting that alternative extraction protocols with reduced amounts of solvents and energy can provide alginate of high quality.

### 3.4. FTIR/ATR

**Figure 6:**
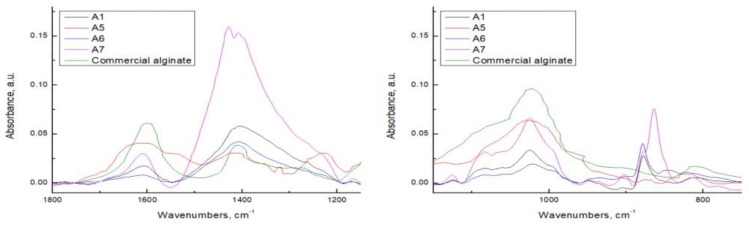
FTIR/ATR spectra of alginates.

The FTIR/ATR spectra of extracted alginates by MAE under dynamic conditions are presented in Figure 6 and compared with the FTIR/ATR spectrum of commercial alginate. The FTIR spectra of alginates extracted under static conditions are not presented, because of the overlapped peaks and no significant changes in comparison to the corresponding alginates obtained by MAE under dynamic conditions. From the analysis of the curves, it is worth highlighting that all the extractive methods used in this work are effective in the releasing of sodium alginate from cell tissue. In fact, similar peaks related to asymmetric (1600 to 1610 cm^−1^) and symmetric (1400–1410 cm^−1^) stretching vibration of carboxylate O–C–O sodium alginate group can be detected in the spectra of all extracted alginates [29,46]. The intensity of these peaks is mostly pronounced in A4 and A7 samples, as expected considering that the alkaline extractive phase allows the solubilization of alginic acid in the form of carboxylated groups. Moreover, the peaks in the region between 1100 and 1000 cm^−1^ related to C-C, C-O and C-O-C stretching vibrations of the pyranose ring are mostly marked in A4 and A7 samples.

A particularly interesting area for analysis of polysaccharides is the anomeric region between 950 and 700 cm^−1^. Generally, alginate displays two peaks in this region, one around 810 cm^−1^ and one as a small shoulder peak around 780 cm^−1^ corresponding to mannuronic and guluronic acid, respectively [47]; these peaks are also detected in the spectrum of commercial alginate. Regarding extracted alginates, these two peaks are noticed only in FTIR spectra of A1, A2, A4, A5 and A7 samples but at a significantly reduced intensity, in comparison to the commercial alginate. In addition, the shoulder peaks of small intensities at 950 cm^−1^ and shoulder peak at 890 cm^−1^ are found in the FTIR spectrum of commercial alginate, which is ascribed to the C-O stretching vibration of uronic acid and of C1–H deformation vibration of mannuronic acid, respectively [48,49,50]. The peak at 950 cm^−1^ is detected in all spectra of extracted alginates, and an additional peak of small intensity at 905 cm^−1^ related to the α-l-guluronic asymmetric ring vibration can be seen, too [49]. It is interesting to note that a narrow peak of strong intensity is located at 870–880 cm^−1^ in spectra of all extracted alginates, except in the case of A2 and A5 (hot water extraction). Although at that IR frequency is usually C1-H deformation vibration of β-mannuronic acid, the change of peak shape from wide shoulder to narrow peak, and peak intensity from weak to strong, indicates that this is a new peak related to another functional group in alginate. It is found in the literature that peaks at 870–880 cm^−1^ are typical of carbonate ions vibrations [51,52]. Hence, it is assumed that the peak detected in that area mostly comes from the extent content of carbonate ions, with no or minimum contribution of depolymerization reaction of alginate, since there was no repetitive process of purification of extracted alginates. This result agrees with the results obtained by GPC because some fractions of carbonates are detected, too.

Regarding the hot water extraction, the corresponding samples, A2 and A5, evidence two stretching vibrational groups not detected in other samples; specifically, it is possible to note a broad absorption peak around 1650 cm^−1^ overlapping with the carboxylate asymmetric vibrational peak and another broad stretching mode at 1550 cm^−1^, corresponding to amide I and amide II protein vibrations, respectively. Finally, a broad peak at 1250 cm^−1^ was ascribable to the asymmetric stretching vibration of the S=O group [40,53]. This outcome suggests that by use of microwave pre-treatment and hot water extractive method, besides alginate, some proteins and sulfated polysaccharides (such as fucoidan) could be solubilized as well. Finalized to agricultural mulching application, the presence of protein, as a nitrogen source, could represent an upgrading for the agronomic performance of films. Overall, FTIR/ATR analysis reveals that the use of different extraction methods causes changes in intensities of characteristic peaks, but not significant shifts in these peaks, thus indicating that chemical structure does not change subsequently.

### 3.5. Thermal Analysis

**Figure 7:**
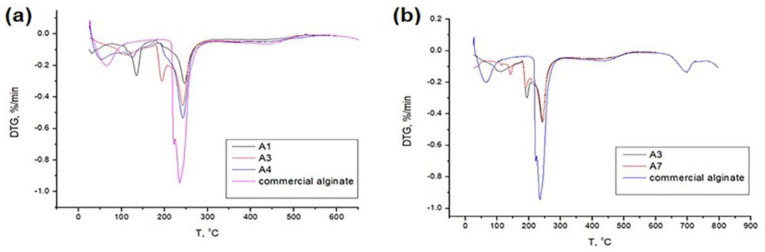
(**a**,**b**) DTG of extracted alginates.

TGA analysis of extracted alginates was performed, and their thermal behavior was compared with the one of commercial alginate. Considering the very low sodium alginate extraction yield of A2 and A5 samples and their complex structure, these samples have not been considered in this analysis. In order to better visualize the thermal degradation pattern and kinetics of selected samples, only DTG thermograms are shown (Figure 7), whereas all corresponding parameters related to the degradation of samples are detailed in Table 3.

The thermal degradation of commercial alginate occurs in two main steps: the first one up to 100 °C corresponds to the evaporation of free water, and the second step, starting at 215 °C and peaking at 235 °C, is related to the decomposition process of alginate, which involves a random split of the glycosidic bonds, vaporization and elimination of volatile products [54]. All extracted alginates show the above-mentioned degradation steps during the heating up to 600 °C, but also evidence of one more degradation step in the range between 100 °C and 180 °C. The weight loss in this region is around 15% for all samples and can be associated with the releasing of bound water [55], the dehydration of extent carbonate ions in the samples [56] and the degradation of some polyphenolic and/or protein fractions [41]. It is hard to connect changes in this region with the variation in extraction methods since the many complex degradation patterns are at the expense of several different polymer and molecule fractions. On the other side, the onset degradation temperature of *Sargassum*-derived alginates is shifted to lower values ranging between 27 and 35 °C, if compared with commercial alginate. The highest shift is detected for all samples extracted by the conventional method. It is interesting to note that DTG diagrams of A4 and A7 samples display a wide shoulder peak in the region between 180 and 330 °C, whereas for the A3 and A6 samples doublet peak appears and is probably related to the degradation of mannuronic residues at lower temperature, which is subsequently followed by degradation of guluronic units. This result confirms that microwave pre-treatment combined with the conventional extractive method causes partial hydrolysis of alginate. The double peak during the thermal degradation of extracted alginate by the acid+alkaline method is supported by literature data [57]. Although the T_onset_ temperature is significantly lower for all extracts, the maximum thermal degradation of alginate macromolecular chains is delayed by 10 °C, in comparison to the commercial alginate. In addition, there are no significant differences (i.e., temperature shifts) in degradation pattern between two used microwave pre-treatments samples. The only visible difference is that weight loss rate for the A6 and A7 (microwave dynamic conditions) is lower than for the A3 and A4 (microwave static conditions), which is evidenced in reduced peak intensity in the region between 180 and 330 °C. This result matches well with spectroscopic (FTIR-ATR) and molecular (GPC) analysis.

### 3.6. Potential Application

**Figure 8:**
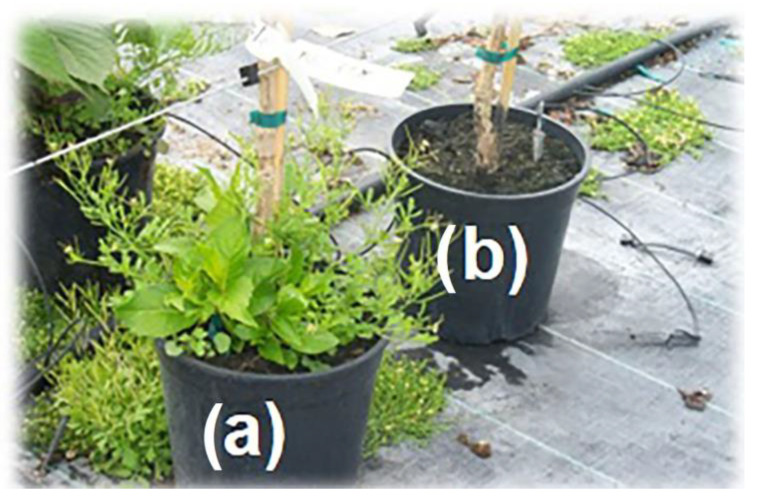
(**a**) untreated and (**b**) treated *Hortensia Dienamann* after 90 days.

In order to evaluate the potential applications of alginate extracted from *Sargassum* seaweed wastes, preliminary tests have been performed using them as mulching geo-membrane on *Hortensia Dienamann* (HD) plant. Mulching polysaccharide-based films have gained increased attention in agriculture to minimize weed appearance [58]. Namely, weed represents one of the major issues in plant cultivation, due to competition for the plant resources and attracting crop pests. The negative effects of uncontrolled growth of weeds are more pronounced in container cultivations than in field cultivations, due to the reduced availability of growing media. In this work, the A7 formulation was dissolved in water at 1 wt% concentration and sprayed on an HD pot and left for 90 days at 23 °C. The HD pot was irrigated only with water and stored in the same conditions as alginate sprayed HD pot was used as control. Figure 8 represents the untreated and treated HD pots after 3 months. By comparing the untreated (Figure 8a) and treated (Figure 8b) plants, it is possible to highlight a huge difference in terms of the uncontrolled growth of the weeds. Indeed, the plant sprayed with alginate does not show any infesting weeds, providing a healthy growth of the leaves, whereas untreated pot demonstrates a significantly high level of weed. Up to date alginate oligosaccharides [59,60,61], gamma-irradiated alginates [62,63], and alginates capsules/films as matrices for bacteria/nutrients [64] have proved to be efficient as plant-growth promoters. The preliminary HD pot result obtained in this work suggests that extracted alginates preserving all the proteins, minerals, pigments, and bioactive molecules, can provide a beneficial effect on the healthy growth of crops, without further modification or additional nutrients.

## 4. Conclusions

In this investigation, microwave-assisted extraction of alginate from *Sargassum* algae under static and dynamic conditions using a fluidized bed in a microwave cavity was reported. It was demonstrated that sodium alginate can be successfully extracted after both microwave pre-treatment regimes, using mild extracting conditions. The yield of obtained alginates was in the range between 20 and 36%, depending on extraction solvents. The highest yields were obtained when the conventional protocol was applied (acid+alkaline extractive method), and microwave pre-treatment was followed by the acid+alkaline protocol. The microwave pre-treatment method under dynamic conditions was the most efficient approach to obtain alginates of higher molecular weights (419–458 kDa), as opposed to the conventional methods of untreated algae (382 kDa). Although microwave dynamic treatment followed by alkaline extraction produced lower yields of alginates (20–24%) in comparison to the conventional protocol of untreated algae, this method is more time and energy-efficient (extraction time approximately 3 h, in comparison of 28 h for A1 sample), requiring less amount of solvents to be used for extraction, and providing the alginates of higher molecular weight and higher thermal stability. Overall, results related to sodium alginate extraction from wastes of raw *Sargassum* algae, evidenced that controlled microwave treatment under dynamic conditions could really represent a valid method to obtain sodium alginate in a cost-effective and eco-sustainable method, by providing, at the same time, an environmentally friendly approach finalized to the recovering, collection and upgrading of urban beach raw waste materials.

## Figures and Tables

**Table 1:** The alginate extraction methods, its yield, and identification codes of samples.

Samples	Sample Codes	Extraction Method	Yield, %
Untreated algae	A1	Conventional method	32
MAE treated algae under static conditions	A2	Hot water method	3
MAE treated algae under static conditions	A3	Conventional method	32
MAE treated algae under static conditions	A4	Alkaline method	20
MAE treated algae under dynamic conditions	A5	Hot water method	1
MAE treated algae under dynamic conditions	A6	Conventional method	36
MAE treated algae under dynamic conditions	A7	Alkaline method	24

**Table 2:** Molecular weight and viscosity of extracted and commercial alginate.

Sample	Mn, Da	Mw, Da	η, dL/g
*A1*	53,232	382,332	6
*A2*	6000	7.5 × 10^6^	3
*A3*	72,673	196,232	4
*A4*	53,477	298,704	7
*A5*	12,672	458,570	2
*A6*	85,221	458,570	7
*A7*	90,518	419,130	6
*Commercial alginate*	48,054	80,520	5

**Table 3:** Thermal parameters of selected alginate samples: Weight loss at 100 °C (W_L100_,%) and 180 °C (W_L180_, %), temperature at which main degradation step starts (T_ONSET_, °C) and temperature at which the maximum degradation rate occurs (T_DEG_, °C).

Sample	W_L100_,%	W_L180_, %	T_onset_, °C	T_deg_, °C
*A1*	6	15	180	246
*A3*	5	13	180	193; 243
*A4*	8	17	188	242
*A6*	5	13	180	192; 242
*A7*	6	16	185	243
*Commercial alginate*	9	13	215	235

## Data Availability

Not available.

## References

[1] https://www.vi.gov.com.

[2] Wang M., Hu C., Barnes B.B., Mitchum G., Lapointe B., Montoya J.P. (2019). The great Atlantic *Sargassum* belt. Science.

[3] Rotter A., Barbier M., Bertoni F., Bones A.M., Cancela M.L., Carlsson J., Carvalho M.F., Cegłowska M., Chirivella-Martorell J., Conk Dalay M. (2021). The Essentials of Marine Biotechnology. Front. Mar. Sci..

[4] Abka-khajouei R., Tounsi L., Shahabi N., Patel A.K., Abdelkafi S., Michaud P. (2022). Structures, Properties and Applications of Alginates. Mar. Drugs.

[5] Pezzoni M., Lemos M., Pizarro R.A., Costa C.S. (2022). UVA as environmental signal for alginate production in Pseudomonas aeruginosa: Role of this polysaccharide in the protection of planktonic cells and biofilms against lethal UVA doses. Photochem. Photobiol. Sci..

[6] Draget K.I., Skjåk-Bræk G., Stokke B.T. (2006). Similarities and differences between alginic acid gels and ionically crosslinked alginate gels. Food Hydrocoll..

[7] Straccia M.C., Romano I., Oliva A., Santagata G., Laurienzo P. (2014). Crosslinker effects on functional properties of alginate/N-succinylchitosan based hydrogels. Carbohydr. Polym..

[8] Immirzi B., Santagata G., Vox G., Schettini E. (2009). Preparation, characterisation and field-testing of a biodegradable sodium alginate-based spray mulch. Biosyst. Eng..

[9] Saji S., Hebden A., Goswami P., Du C. (2022). A Brief Review on the Development of Alginate Extraction Process and Its Sustainability. Sustainability.

[10] Beata Łabowska M., Michalak I., Detyna J. (2019). Methods of extraction, physicochemical properties of alginates and their applications in biomedical field—A review. Open Chem..

[11] Bagherian H., Zokaee Ashtiani F., Fouladitajar A., Mohtashamy M. (2011). Comparisons between conventional, microwave- and ultrasound-assisted methods for extraction of pectin from grapefruit. Chem. Eng. Process. Process Intensif..

[12] Zannini D., Dal Poggetto G., Malinconico M., Santagata G., Immirzi B. (2021). Citrus Pomace Biomass as a Source of Pectin and Lignocellulose Fibers: From Waste to Upgraded Biocomposites for Mulching Applications. Polymers.

[13] Yuan Y., Macquarrie D.J. (2015). Microwave assisted step-by-step process for the production of fucoidan, alginate sodium, sugars and biochar from Ascophyllum nodosum through a biorefinery concept. Bioresour. Technol..

[14] Okolie C.L., Mason B., Mohan A., Pitts N., Udenigwe C.C. (2020). Extraction technology impacts on the structure-function relationship between sodium alginate extracts and their in vitro prebiotic activity. Food Biosci..

[15] Torabi P., Hamdami N., Keramat J. (2022). Microwave-assisted extraction of sodium alginate from brown macroalgae Nizimuddinia zanardini, optimization and physicochemical properties. Sep. Sci. Technol..

[16] McElroy C.R., Kopanitsa L., Helmes R., Fan J., Attard T.M., Simister R., van den Burg S., Ladds G., Bailey D.S., Gomez L.D. (2023). Integrated biorefinery approach to valorise Saccharina latissima biomass: Combined sustainable processing to produce biologically active fucoxanthin, mannitol, fucoidans and alginates. Environ. Technol. Innov..

[17] Michalak I., Tuhy Ł., Chojnacka K. (2015). Seaweed extract by microwave assisted extraction as plant growth biostimulant. Open Chem..

[18] Toan T.Q., Phong T.D., Tien D.D., Linh N.M., Mai Anh N.T., Hong Minh P.T., Duy L.X., Nghi D.H., Pham Thi H.H., Nhut P.T. (2021). Optimization of Microwave-Assisted Extraction of Phlorotannin From *Sargassum swartzii* (Turn.) C. Ag. with Ethanol/Water. Nat. Prod. Commun..

[19] del Río P.G., Gullón B., Pérez-Pérez A., Romaní A., Garrote G. (2021). Microwave hydrothermal processing of the invasive macroalgae *Sargassum muticum* within a green biorefinery scheme. Bioresour. Technol..

[20] Herawati D., Pudjiastuti P., Zaidan A.H., Hendradi E., Wafiroh S. (2022). Fucoidan from *Sargassum plagiophyllum* by Microwave Assisted Extraction in Comparison with Conventional Methods. Rasayan J. Chem..

[21] Ruslan R., Amir A., Wiraningtyas A. (2019). Extraction of Sodium Alginate from *Sargassum* sp. using Microwave-Assisted Extraction (MAE). J. Pure Appl. Chem. Res..

[22] Mujumdar A.S. (2006). Handbook of Industrial Drying.

[23] Lv W., Li D., Lv H., Jin X., Han Q., Su D., Wang Y. (2019). Recent development of microwave fluidization technology for drying of fresh fruits and vegetables. Trends Food Sci. Technol..

[24] Rovito M.A., De Bonis M.V., Ruocco G. (2019). COLDwaveTM processing: Cold jet impingement to control bio-substrate drying by microwave and preserve its quality. Heat Mass Transf..

[25] Pace M., De Bonis M.V., Marra F., Ruocco G. (2011). Characterization of a combination oven prototype: Effects of microwave exposure and enhanced convection to local temperature rise in a moist substrate. Int. Commun. Heat Mass Transf..

[26] Vyazovkin S., Burnham A.K., Criado J.M., Pérez-Maqueda L.A., Popescu C., Sbirrazzuoli N. (2011). ICTAC Kinetics Committee recommendations for performing kinetic computations on thermal analysis data. Thermochim. Acta.

[27] Fertah M., Belfkira A., Dahmane E.M., Taourirte M., Brouillette F. (2017). Extraction and characterization of sodium alginate from Moroccan Laminaria digitata brown seaweed. Arab. J. Chem..

[28] Khajouei R.A., Keramat J., Hamdami N., Ursu A.-V., Delattre C., Laroche C., Gardarin C., Lecerf D., Desbrières J., Djelveh G. (2018). Extraction and characterization of an alginate from the Iranian brown seaweed Nizimuddinia zanardini. Int. J. Biol. Macromol..

[29] Mohammed A., Bissoon R., Bajnath E., Mohammed K., Lee T., Bissram M., John N., Jalsa N.K., Lee K.-Y., Ward K. (2018). Multistage extraction and purification of waste *Sargassum natans* to produce sodium alginate: An optimization approach. Carbohydr. Polym..

[30] Abraham R.E., Su P., Puri M., Raston C.L., Zhang W. (2019). Optimisation of biorefinery production of alginate, fucoidan and laminarin from brown seaweed Durvillaea potatorum. Algal Res..

[31] Rostami Z., Tabarsa M., You S., Rezaei M. (2017). Relationship between molecular weights and biological properties of alginates extracted under different methods from Colpomenia peregrina. Process Biochem..

[32] Andriamanantoanina H., Rinaudo M. (2010). Characterization of the alginates from five madagascan brown algae. Carbohydr. Polym..

[33] Aguilar K.C., Tello F., Bierhalz A.C.K., Garnica Romo M.G., Martínez Flores H.E., Grosso C.R.F. (2015). Protein adsorption onto alginate-pectin microparticles and films produced by ionic gelation. J. Food Eng..

[34] Mazumder A., Holdt S.L., De Francisci D., Alvarado-Morales M., Mishra H.N., Angelidaki I. (2016). Extraction of alginate from *Sargassum muticum*: Process optimization and study of its functional activities. J. Appl. Phycol..

[35] Guolin H., Jeffrey S., Kai Z., Xiaolan H. (2012). Application of Ionic Liquids in the Microwave-Assisted Extraction of Pectin from Lemon Peels. J. Anal. Methods Chem..

[36] Cebrián-Lloret V., Metz M., Martínez-Abad A., Knutsen S.H., Ballance S., López-Rubio A., Martínez-Sanz M. (2022). Valorization of alginate-extracted seaweed biomass for the development of cellulose-based packaging films. Algal Res..

[37] Klinger M. Depolymerization of alginate 2013.

[38] Chen X., Yang J., Shen M., Chen Y., Yu Q., Xie J. (2022). Structure, function and advance application of microwave-treated polysaccharide: A review. Trends Food Sci. Technol..

[39] Yudiati E., Djarod M.S.R., Pringgenies D., Susilo E.S. (2019). Accelerating The Physilogical Properties of Sodium Alginate Paste by Thermal Method and Microwave Irradiation. IOP Conf. Ser. Earth Environ. Sci..

[40] Flórez-Fernández N., Domínguez H., Torres M.D. (2019). A green approach for alginate extraction from *Sargassum muticum* brown seaweed using ultrasound-assisted technique. Int. J. Biol. Macromol..

[41] Santagata G., Grillo G., Immirzi B., Tabasso S., Cravotto G., Malinconico M. (2018). Non-conventional Ultrasound-Assisted Extraction of Alginates from Sargassum seaweed: From Coastal Waste to a Novel Polysaccharide Source. Proceedings of the International Conference on Microplastic Pollution in the Mediterranean Sea.

[42] Nogueira M.T., Chica L.R., Yamashita C., Nunes N.S.S., Moraes I.C.F., Branco C.C.Z., Branco I.G. (2022). Optimal conditions for alkaline treatment of alginate extraction from the brown seaweed *Sargassum cymosum* C. Agardh by response surface methodology. Appl. Food Res..

[43] Rahelivao M.P., Andriamanantoanina H., Heyraud A., Rinaudo M. (2013). Structure and properties of three alginates from Madagascar seacoast algae. Food Hydrocoll..

[44] Torres M.R., Sousa A.P.A., Silva Filho E.A.T., Melo D.F., Feitosa J.P.A., de Paula R.C.M., Lima M.G.S. (2007). Extraction and physicochemical characterization of *Sargassum vulgare* alginate from Brazil. Carbohydr. Res..

[45] Larsen B., Salem D.M.S.A., Sallam M.A.E., Mishrikey M.M., Beltagy A.I. (2003). Characterization of the alginates from algae harvested at the Egyptian Red Sea coast. Carbohydr. Res..

[46] Mohammed A., Rivers A., Stuckey D.C., Ward K. (2020). Alginate extraction from *Sargassum* seaweed in the Caribbean region: Optimization using response surface methodology. Carbohydr. Polym..

[47] Mackie W. (1971). Semi-quantitative estimation of the composition of alginates by infra-red spectroscopy. Carbohydr. Res..

[48] Leal D., Matsuhiro B., Rossi M., Caruso F. (2008). FT-IR spectra of alginic acid block fractions in three species of brown seaweeds. Carbohydr. Res..

[49] Fawzy M.A., Gomaa M., Hifney A.F., Abdel-Gawad K.M. (2017). Optimization of alginate alkaline extraction technology from *Sargassum latifolium* and its potential antioxidant and emulsifying properties. Carbohydr. Polym..

[50] Chandía N. (2001). Alginic acids in Lessonia trabeculata: Characterization by formic acid hydrolysis and FT-IR spectroscopy. Carbohydr. Polym..

[51] Fleet M.E. (2009). Infrared spectra of carbonate apatites: ν2-Region bands. Biomaterials.

[52] Database of ATR-FT-IR Spectra of Various Materials. https://spectra.chem.ut.ee/.

[53] Derkach S.R., Voron’ko N.G., Kuchina Y.A. (2022). Intermolecular Interactions in the Formation of Polysaccharide-Gelatin Complexes: A Spectroscopic Study. Polymers.

[54] Nešić A., Onjia A., Davidović S., Dimitrijević S., Errico M.E., Santagata G., Malinconico M. (2017). Design of pectin-sodium alginate based films for potential healthcare application: Study of chemico-physical interactions between the components of films and assessment of their antimicrobial activity. Carbohydr. Polym..

[55] Russo R., Abbate M., Malinconico M., Santagata G. (2010). Effect of polyglycerol and the crosslinking on the physical properties of a blend alginate-hydroxyethylcellulose. Carbohydr. Polym..

[56] Siriwardane R.V., Poston J.A., Robinson C., Simonyi T. (2011). Effect of Additives on Decomposition of Sodium Carbonate: Precombustion CO_2_ Capture Sorbent Regeneration. Energy Fuels.

[57] Torres M.L., Cortizo A.M., Oberti T.G., Fernandez J.M. (2016). Characterization of Commercial and Algae (*Undaria pinnatifida*) Extracted Sodium Alginate for Future Application in Bone Tissue Engineering. Environ. Sci..

[58] Giaccone M., Cirillo C., Scognamiglio P., Teobaldelli M., Mataffo A., Stinca A., Pannico A., Immirzi B., Santagata G., Malinconico M. (2018). Biodegradable mulching spray for weed control in the cultivation of containerized ornamental shrubs. Chem. Biol. Technol. Agric..

[59] Li Z., Duan S., Lu B., Yang C., Ding H., Shen H. (2023). Spraying alginate oligosaccharide improves photosynthetic performance and sugar accumulation in citrus by regulating antioxidant system and related gene expression. Front. Plant Sci..

[60] Salachna P., Grzeszczuk M., Meller E., Soból M. (2018). Oligo-Alginate with Low Molecular Mass Improves Growth and Physiological Activity of *Eucomis autumnalis* under Salinity Stress. Molecules.

[61] González A., Castro J., Vera J., Moenne A. (2013). Seaweed Oligosaccharides Stimulate Plant Growth by Enhancing Carbon and Nitrogen Assimilation, Basal Metabolism, and Cell Division. J. Plant Growth Regul..

[62] Hossain M.A., Islam J.M.M., Hoque M.M., Nahar S., Khan M.A. (2021). Field demonstration of irradiated sodium alginate as tea production booster. Heliyon.

[63] El-Mohdy H.L.A. (2017). Radiation-induced degradation of sodium alginate and its plant growth promotion effect. Arab. J. Chem..

[64] Martínez-Cano B., Mendoza-Meneses C.J., García-Trejo J.F., Macías-Bobadilla G., Aguirre-Becerra H., Soto-Zarazúa G.M., Feregrino-Pérez A.A. (2022). Review and Perspectives of the Use of Alginate as a Polymer Matrix for Microorganisms Applied in Agro-Industry. Molecules.

